# Multi-Functional Hypercrosslinked Polystyrene as High-Performance Adsorbents for Artificial Liver Blood Purification

**DOI:** 10.3389/fchem.2021.789814

**Published:** 2022-01-17

**Authors:** Yunhong Liu, Xinyan Peng

**Affiliations:** College of Chemical Engineering and Materials Science, Quanzhou Normal University, Quanzhou, China

**Keywords:** hemoperfusion, adsorptive resin, hemocompatibility, artificial liver blood purification, hypercrosslinked polystyrene

## Abstract

In artificial liver blood purification system, highly efficient removal of multiple toxic metabolites from whole blood by hemoperfusion still remains a challenge in the clinical field, due to the limited unspecific absorptive capacity and low biocompatibility of adsorbents. In this work, a new pyridinyl-modified hypercrosslinked polystyrene (HCP) adsorbent, named HCP(St-DVB-VP), was constructed directly through a Friedel-Crafts post-crosslinking reaction using a small-molecule crosslinking agent for the first time. The preparation method provides in this study can avert the problem posed by the use of the toxic carcinogenic chloromethyl ether reagent in the traditional HCP resin synthesis process. The results indicated that HCP(St-DVB-VP) had a highly porous structure with a specific surface area of 761 m^2^ g^−1^. Notably, the adsorbent demonstrated excellent adsorptive properties towards both protein-bound toxins (bilirubin) and medium- and large-sized molecular toxins (PTH, IL-6) *in vitro* experiments simultaneously. More importantly, the obtained adsorbent showed acceptable hemocompatibility. Taken together, the low-cost and ecofriendly fabrication method, broad-spectrum adsorption performance and hemocompatibility makes the HCP(St-DVB-VP) promising for whole blood perfusion in artificial liver blood purification in clinical practice.

## 1 Introduction

Liver failure is the inability of the liver to perform its normal synthetic, metabolic, excretory and biotransformation functions. For patients with advanced liver disease, the accumulation of toxic metabolites results in a series of serious clinical consequences. These toxic substances principally include protein-bound toxins (like bilirubin) and medium- and large-sized molecular toxins (like cytokines), etc. These toxins can cause local injuries and systemic injuries, such as systemic inflammatory response syndrome and multiple organ failure. Effectively removing these toxic substances from the blood is a fundamental goal of the application of artificial liver for the treatment of liver failure (Hansen, 2002; Li, 2017). According to the treatment experience of severe COVID-19 patients, artificial liver blood purification treatment has proven effective at significantly blocking the cytokine storm and supporting liver function (Channappanavar and Perlman, 2017; Dai et al., 2021). The artificial liver support system (ALSS) is a body circulatory system aims to blood purification, which consists of plasma exchange, hemodialysis, hemoperfusion, in combination with hemodiafiltration (Jiang et al., 2009).

Hemoperfusion is a process in which blood is passed through an adsorbent system to remove toxic substances from the blood. The commonly used adsorbents are activated carbon (AC) and resin which includes ion-exchangers, high surface area adsorbents, etc. (Chen et al., 2017a; Ju et al., 2019; Yamamoto et al., 2019; Liu and Peng, 2021). However, these existing adsorbents was found to have limited unspecific absorptive capacity and poor biocompatibility. For example, plasma perfusion with ion exchange resins (e.g., BS330 from Jafron, China, and BR350 from Asahi Kasei Medical, Japan) was applied for the treatment of hyperbilirubinemia through a strong electrostatic force between negatively charged bilirubin and the high density of quaternary ammonium group (Adani et al., 2007; Meijers et al., 2007). However, strong anion exchange resins show dissatisfactory hemocompatibility, and a large dose of injected heparin leads to the increasing cost and the risk of clinical treatment.

In terms of medium- and large-sized molecular toxins, crosslinked polystyrene adsorption resins could be a good choice, such as CytoSorb™ cartridge (Cytosorbents, NJ, United States) and HA series hemoperfusion cartridge (Jafron, China). The CytoSorb adsorbent has been proven to be efficient in removing excessive cytokines from the blood in severe sepsis (Morris et al., 2015). Yet, due to a large average pore diameter of the adsorbent, it exhibits obvious nonspecific adsorption to albumin. As a hyper-crosslinked polystyrenes adsorbent, HA resin has abundant micropores and high specific surface area, exhibiting excellent adsorption properties for large middle molecular weight toxins (parathyroid hormone, b2-microglobulin, cytokines, etc.) (Ankawi et al., 2019; Lorenzin et al., 2019). In addition, HA resin possesses outstanding hydrophilicity and blood compatibility, making it suitable for whole blood perfusion. Nevertheless, they also have some significant shortcomings, such as inadequate removal of protein-bound toxins and the lack of sufficiently advanced preparation methods. The hypercrosslinked polystyrene adsorptive resin for hemoperfusion are mainly obtained through a Friedel-Crafts post-crosslinking reaction after chloromethylation of a macroporous hypocrosslinked polystyrene-divinylbenzene copolymer (Joseph et al., 2015). The synthesis process is not only cumbersome and complicated but also causes certain harm to the environment and human health due to the use of toxic and carcinogenic chemicals, such as chloromethyl ether. In addition, the production cost is high. Therefore, the synthesis process needs further technical optimization and improvement.

Indeed, the substances needed to be removed through artificial liver blood purification treatment are a complex mixture, and the composition has not been completely identified. Due to the limited and unspecific absorptive capacity of the existing adsorbent, it is difficult to clear toxins from the blood-stream by direct hemoperfusion using an adsorbent with single adsorption characteristics. Some researches demonstrated that the combination of two kinds of adsorbents may allow their respective adsorption characteristics into full play. In clinical practice, the dual plasma molecular adsorption system (DPMAS) that comprising a neutral macroporous resin (HA330-II from Jafron, China) and ion exchange resin (BS330 from Jafron, China), was applied for the treatment of patients with clinically liver failure (Yang et al., 2021). This typical non‐biological artificial liver system may achieve simultaneous removal of bilirubin and inflammation medium, achieving a synergistic blood-purifying effect. The DPMAS system can block cytokine storms and functionally support liver function, reduce inflammatory reactions and toxin-induced damage to the body, so it has been used in the treatment of coronavirus disease 2019 (COVID-19) (Wang and Hu, 2020), as shown in Supplementary Figure S1. However, this system uses multiple separators/adsorbers and involves a plasma adsorption process that requires complicated pipe/hose network and devices, causing high treatment cost; as a result, it is not suitable for large-scale use in clinical practice.

The aim of the present study was to prepare a multi-functional adsorbent, integrating the blood-purifying function of the neutral macroporous resin and ion exchange resin. The adsorbent is a novel functional pyridinyl-containing hypercrosslinked polystyrene resin (HCP(St-DVB-VP)) prepared through Friedel-Crafts post-crosslinking using a small-molecular-weight crosslinking agent. It can simultaneously eliminate a variety of toxins with high efficiency, including protein-bound toxins (bilirubin) and medium- and large-sized molecular toxins (PTH, IL-6) to achieve a broad adsorption effect, which showed the similar adsorption property with DPMAS, while the cost is much cheaper. In addition, HCP(St-DVB-VP) adsorbent exhibited a low hemolysis rate and low clotting risk *in vitro*. The broad-spectrum adsorption performance, acceptable hemocompatibility and cost-effective of HCP(St-DVB-VP) make it promising for whole blood perfusion in artificial liver blood purification treatments.

## 2 Experiment

### 2.1 Reagents and Instruments

Styrene (St, 99%), 4-vinylpyridine (VP, 96%), benzoyl peroxide (BPO, analytical reagent (AR) grade), trimethyl orthoformate (TMOF, 98%), bilirubin (98%), bile acid (98%), lysozyme (≥20,000 U/mg, from egg white), and anhydrous ferric chloride (AR grade) were purchased from Shanghai Aladdin Bio-Chem Technology Co., Ltd. Divinylbenzene (DVB, 80%) was purchased from Jiangsu Haolong Chemical Co., Ltd. Gelatine (99%), methyl isobutyl carbinol (MIBC, 99%), 1,2-dichloroethane (DCE, AR grade), and phosphate-buffered saline (PBS, pH 7.4) were purchased from Shanghai Macklin Biochemical Co., Ltd. Ethanol (95%), sodium chloride (AR grade), and magnesium chloride (AR grade) were purchased from Sinopharm Chemical Reagent Co., Ltd. Parathyroid hormone (PTH) and IL-6 were purchased from R&D Systems, United States. All reagents were used as received.

The chemical group composition on the material surface was analysed via Fourier-transform infrared spectroscopy (FTIR) (Nicolet iS50, Thermo Fisher Scientific, United States). Field emission scanning electron microscopy (SEM) (Model Merlin, Zeiss, Germany) was used to observe the microtopographic features of the samples. The chemical element composition and valence state of the sample surface were analysed via X-ray photoelectron spectroscopy (JPS-9200, JEOL Corporation, Japan). The specific surface area and porous structure of the materials were analysed using a 3Flex surface characterization analyser (Micromeritics, United States). Toxin concentrations were detected with an ultraviolet-visible (UV-Vis) spectrometer.

### 2.2 Experimental Procedure

#### 2.2.1 Synthesis of HCP Adsorptive Resin

##### 2.2.1.1 Preparation of Pyridinyl-Modified Hypocrosslinked Polystyrene Porous Resin

The suspension polymerization method was adopted. First, after complete dissolution of the gelatine dispersant by stirring 200 ml of deionized water and 4 g of gelatine in a 500 ml three-mouth flask for 8 h at room temperature, 4 g of sodium chloride and 5 g of magnesium chloride were sequentially added and stirred to obtain an aqueous phase. Second, the comonomers (29 g St, 5.8 g DVB, and 2 g VP), initiator (0.6 g BPO), and porogens (5 g toluene and 45 g MIBC) were weighed and mixed at room temperature until the initiator was completely dissolved to obtain an oil phase. Subsequently, the oil phase was added to the aqueous phase. After reaction at 78°C for 8 h, the mixture was heated to 85°C for a 3 h reaction. Finally, after polymerization was completed, the mixture was washed with water and 95% ethanol several times successively to remove dispersant and porogens and then dried to obtain pyridinyl-modified hypocrosslinked polystyrene microspheres, which were named P(St-DVB-VP), where St-DVB-VP stands for styrene–divinylbenzene-4-vinylpyridine.

##### 2.2.1.2 Preparation of Pyridyl-Modified HCP Adsorption Resin

First, 18 g of pyridinyl-modified hypocrosslinked pyridinyl-modified polystyrene porous resin and 300 ml of dichloroethane were added into a three-mouth flask and stirred at room temperature for 12 h to make the resin fully swollen. Second, 140 g of TMOF and 108 g of anhydrous ferric chloride were sequentially added and reacted at 50°C for 3 h, and then, the temperature was raised to 80–83°C to continue the reaction for 8 h. Third, after the resin was kept still and cooled, it was separated and washed with ethanol until turning clear. Fourth, the resin was washed in hydrochloric acid solution (5%) for 4 h and then washed with water until reaching a neutral pH (pH ≈ 7). Finally, after being extracted with ethanol in a Soxhlet extractor for 8 h and oven-dried, the pyridyl-modified HCP adsorptive resin was obtained and named HCP(St-DVB-VP).

#### 2.2.2 Adsorptive Property Tests

##### 2.2.2.1 Adsorptive Property Analysis Towards Large Molecular-Sized Toxins in Human Plasma Environment

The adsorption capacity of the material towards proteins and medium- and large-sized molecular toxins, such as IL-6 and PTH, was tested with human plasma as the testing environment. First, PTH and IL-6 were added into human plasma to prepare toxin-containing plasma solutions with PTH and IL-6 concentrations of 180 pmol L^−1^ and 300 pg ml^−1^, respectively. Second, 1 ml of dry resin (HA330-Ⅱ: 0.23 g, BS330: 0.31 g, HCP(St-DVB-VP): 0.25 g) was measured and placed into a 25 ml triangular flask and then infiltrated with PBS for 12 h. After the resin was blotted dry, 10 ml of toxin-containing plasma solution was added into the flask, which was then shaken at 110 rpm and 37°C on a shaker for 2 h. The supernatant solutions before and after adsorption were collected and tested for IL-6, PTH, albumin, and total protein concentrations using the chemiluminescence method, the electrochemiluminescence method, the bromocresol green (BCG) method, and the biuret method, respectively, with the toxin-containing plasma solution without addition of resin as the blank control. The adsorption rate *R* was calculated using the concentration difference of the solute before and after adsorption, as shown in Eq. 1:

$$\text{Adsorption rate }\text{R}\;=\;\frac{C_{0}-C_{S}}{C_{0}}\;\times \;100\text{\%,}$$ (1)

where *R* represents the adsorption rate of the toxin or protein, expressed in %; *C*_*0*_ represents the initial concentration of the toxins; and *Cs* represents the concentration of the toxins after adsorption.

##### 2.2.2.2 Analysis of the Resin Adsorptive Property Towards Bilirubin and Bile Acid in Human Plasma Environment

The adsorption of bilirubin and bile acid by the proposed resin were evaluated using human plasma as the testing environment. First, bilirubin and bile acid were added into human plasma to prepare toxin-containing plasma solutions with bilirubin and bile acid concentrations of 300 μmol/L and 150 μmol/L, respectively. Next, 1 ml of dry resin was measured and placed into a 25 ml triangular flask and then infiltrated with PBS for 12 h. Next, after the excessive PBS solution was blotted away, 10 ml of toxin-containing plasma solution was added into the flask, which was then shaken at 110 rpm and 37°C on a shaker for 2 h. Finally, the solutions collected before and after adsorption were tested for bilirubin and bile acid concentrations using the diazo and velocity method, respectively, and the adsorption rate was calculated. All the above procedures were performed in the dark.

##### 2.2.2.3 Adsorptive Property Analysis Towards Protein in Human Plasma Environment

Adsorption behavior of the resins for albumin and total protein were studied using human plasma as the testing environment. First, 1 ml of dry resin was measured and placed into a 25 ml triangular flask and then infiltrated with PBS for 12 h. Next, after the excessive PBS solution was blotted away, 10 ml of plasma solution was added into the flask, which was then shaken at 110 rpm and 37°C on a shaker for 2 h. Finally, the levels of albumin and total protein in human plasma after treatment with adsorbent resin were detected via the bromocresol green (BCG) method and the biurea method, respectively, and the adsorption rate was calculated.

##### 2.2.2.4 Adsorption Equilibrium and Kinetics of Bilirubin and Lysozyme Adsorption in a PBS Environment

To test the adsorption equilibrium, first, 0.06 g of dry resin was accurately weighed and placed into a 50 ml stoppered conical flask and washed several times with PBS. Next, 20 ml of bilirubin or lysozyme solution with an initial concentration *C*_*0*_ (mg L^−1^) was measured and added into the flask, which was then shaken at 110 rpm and 37°C on a shaker. After the adsorption equilibrium was reached, the absorbance of bilirubin or lysozyme was measured at wavelengths of 438 and 222 nm using a UV-Vis spectrophotometer, respectively. The data were recorded, and the equilibrium concentration *Ce* (mg L^−1^) was calculated. The equilibrium adsorption amount *q*_*e*_ (mg g^−1^) was calculated as follows:

$${\text{q}}_{e}=\frac{\left(C_{0}-C_{e}\right)\times V}{m},$$ (2)

where *V* is the volume of the adsorption solution (ml), and *m* is the mass of the adsorption resin (g).

To assess adsorption kinetics, first, bilirubin solution at 25 mg L^−1^ and lysozyme solution at 50 mg L^−1^ were prepared using PBS. Second, 0.15 g of resin (dry weight) was accurately weighed and placed in a 100 ml stoppered conical flask and washed several times with PBS, followed by addition of 50 ml of bilirubin or lysozyme solution. The flask was shaken at 110 rpm and 37°C on a shaker, during which 0.5 ml of solution was collected regularly for measurement of the absorbance of bilirubin or lysozyme using a UV-Vis spectrophotometer. The readings were recorded and used for concentration calculation. The *q*_*t*_-*t* curve, namely, the adsorption kinetic curve, was generated by plotting the adsorption amount qt (mg g^−1^) at time *t* versus the adsorption time *t* (min). All the above procedures were performed in the dark.

#### 2.2.3 Hemocompatibility Assay

##### 2.2.3.1 Hemolysis Assay

According to previous studies, three sample groups were included in the hemolysis experiment, namely, an experimental group, negative control group, and positive control group. The experimental sample contained 50 mg of resin sample and 10 ml PBS (pH = 7.4) in a centrifuge tube. Each resin type was tested in triplicate. The negative control containing only 10 ml of PBS (pH = 7.4) was tested in triplicate. The positive control containing only 10 ml of distilled water was tested in triplicate. First, 8 ml of fresh rabbit blood was mixed and diluted with 10 ml of PBS to obtain an erythrocyte suspension. Second, 0.3 ml of erythrocyte suspension was added to a centrifuge tube containing a sample from one of the three groups, which were then incubated at 37°C for 1 h. Third, after the samples were centrifuged at 4,000 rpm for 5 min, the supernatant was collected using a pipette and placed into a UV-Vis spectrophotometer. The absorbance of each supernatant was measured at 545 nm, with PBS as the reference solution. The data were recorded, and the hemolysis rate was calculated. The hemolysis rate (*HR*) of the adsorptive resin was calculated using Eq. 3:

$$HR\left(\%\right)=\frac{\left(A_{S}-A_{NC}\right)}{\left(A_{PC}-A_{NC}\right)}\times 100\%,$$ (3)

where *As*, *A*_*NC*_, and *A*_*PC*_ are the absorbances of the samples in the experimental group, the negative control group, and the positive control group, respectively.

##### 2.2.3.2 Recalcification Time Measurement

The first step was sample preparation. The resin sample was placed in a conical flask and normal saline was added. After the resin was fully infiltrated, 0.3 ml of resin was placed in an EP tube and blotted dry for subsequent use. Each sample was tested in triplicate. A blank tube without resin was used as a negative control, and a tube with glass beads was used as a positive control. The second step was to prepare platelet-rich plasma (PRP). Fresh rabbit blood was harvested using an vacuum blood collection tube that contained sodium citrate as an anticoagulant and centrifuged at 1,200 rpm for 20 min. After centrifugation, the supernatant was removed with a pipette to obtain PRP. The third step was contact activation of plasma coagulation. To each sample, 0.6 ml of PRP was added, and the samples were incubated at 37°C for 15 min. During incubation, the sample tubes were turned upside down once every 5 min to ensure full contact of sample with plasma. Subsequently, 100 μl of calcium chloride solution (0.2 mol/L) was simultaneously added to each sample with a multichannel pipette, and a stopwatch was used to start timing. Meanwhile, the sample was placed in a 37°C water bath. The time from the beginning of calcium chloride addition to the complete coagulation of plasma was considered the recalcification time of the resin.

## 3 Results and Discussion

### 3.1 HCP(St-DVB-VP) Preparation Method

**FIGURE 1:**
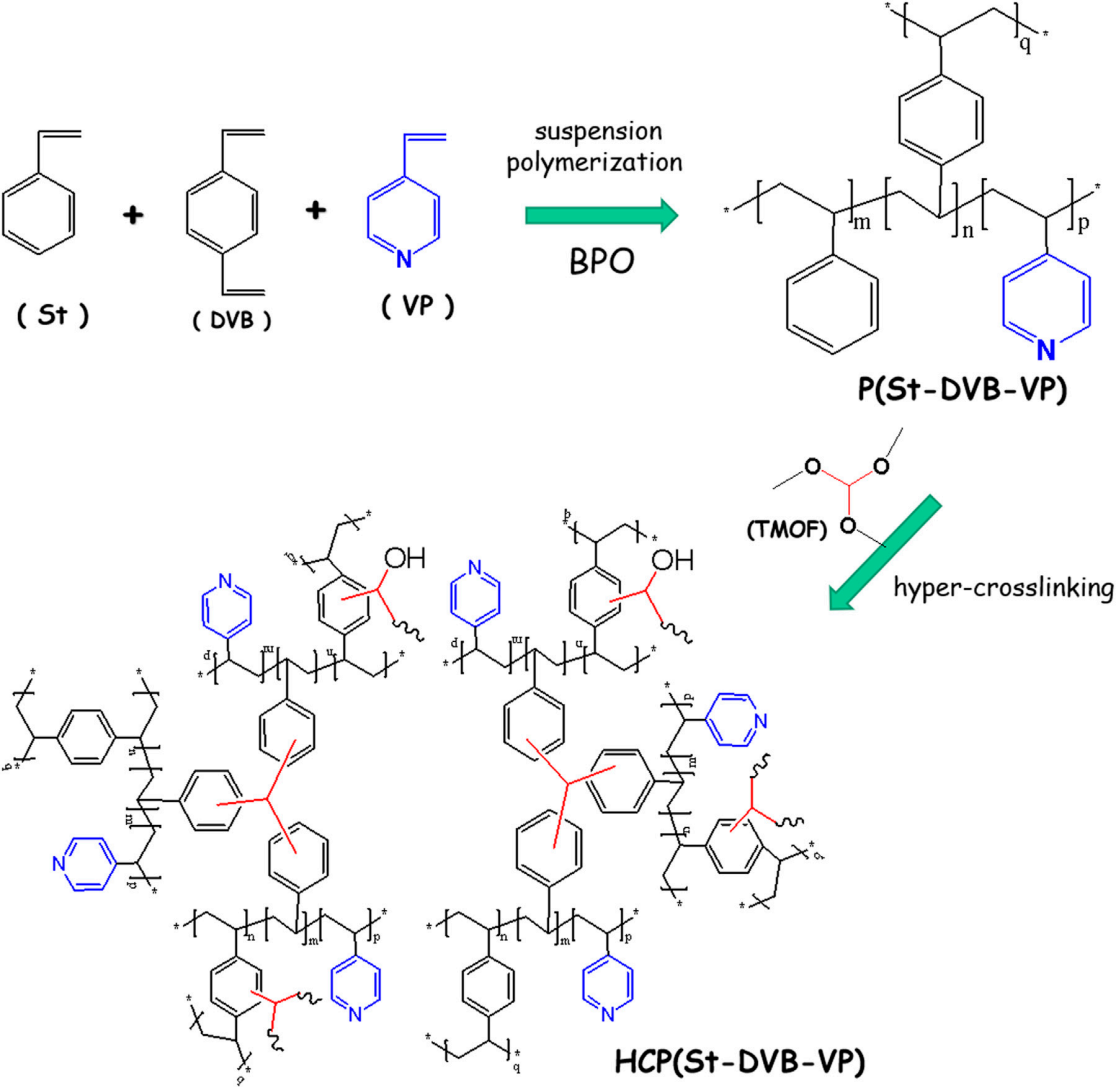
Schematic diagram of the HCP(St-DVB-VMP) fabrication route.

As shown in Figure 1, pyridinyl-modified hypocrosslinked polystyrene microspheres P(St-DVB-VP) were first prepared by copolymerization of St, DVB, and VP via a traditional suspension polymerization method. Subsequently, in the presence of ferric chloride as a catalyst, TMOF was used as a crosslinking agent to enable a complex Friedel-Crafts post-crosslinking reaction between the methoxy group of TMOF and the benzene ring of P(St-DVB-VP), forming a rigid highly crosslinked network (Tan and Tan, 2017). Through this approach, HCP (St-DVB-VP) with a high specific surface area and rich porous structure was efficiently synthesized in one step.

Traditionally, HCP adsorptive resin is obtained by first preparing macroporous hypocrosslinked polystyrene-DVB copolymer and then sequentially subjecting it to chloromethylation and a post-crosslinking reaction (Tsyurupa and Davankov, 2006). Similarly, preparation of strong alkaline polystyrene anion-exchange resin requires sequential chloromethylation and amination of the hypocrosslinked polystyrene-DVB copolymer (Abbasian et al., 2011). The reaction process is cumbersome and involves strong carcinogenic or toxic reagents, such as chloromethyl ether, which will inevitably harm workers and the environment during production. In addition, the production cost is high, and thus, further technical optimization and improvement are required. In this study, the small-molecule TMOF crosslinking agent directly resulted in a post-crosslinking reaction of macroporous hypocrosslinked polystyrene-DVB copolymer with functional groups, thus avoiding the use of the toxic carcinogenic chloromethyl ether reagent used in the traditional HCP resin synthesis process. The method proposed in this study provides a new low-cost, environmentally friendly, and high-efficiency strategy for preparation of functional HCP resin for hemoperfusion.

### 3.2 Microstructural Characterization of Adsorptive Resins

**FIGURE 2:**
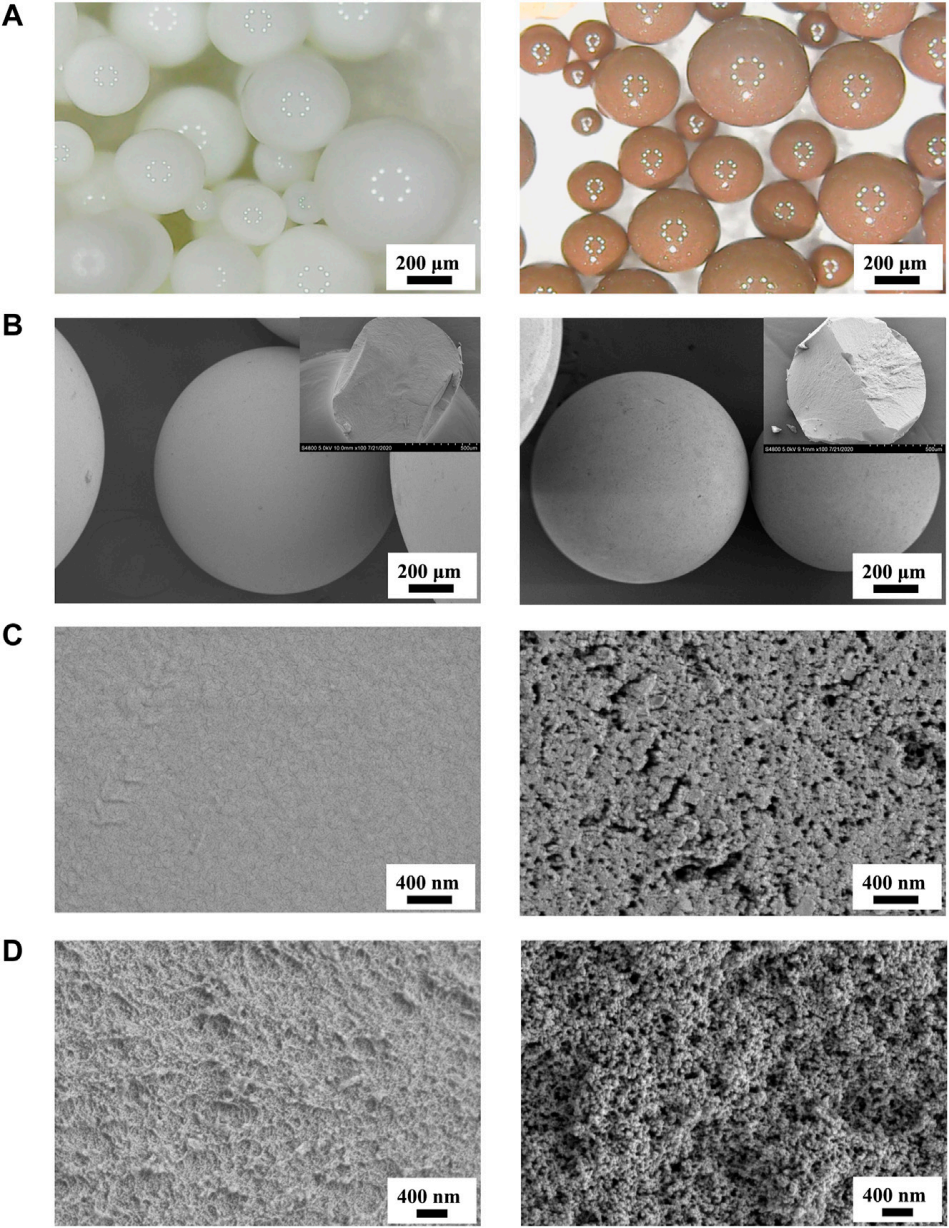
Optical microscopy micrographs **(A)** and SEM images **(B–D)** of P(St-DVB-VP) and HCP(St-DVB-VP). **(C)** The surface zone; **(D)** the cross-sectionn zone. Insets of **(B)** are their SEM cross-section morphologies after mechanical compression. Left: P(St-DVB-VP), right: HCP(St-DVB-VP).

Figure 2 presents optical photographs and SEM photographs of the P(St-DVB-VP) and HCP(St-DVB-VP) resin samples. As seen from Figure 2A, both samples consist of smooth spherical particles with a particle size ranging from 100 to 500 μm. P(St-DVB-VP) is originally white but turns brown once it is transformed to HCP(St-DVB-VP) after the post-crosslinking reaction. The experimental observations revealed that uncrosslinked P(St-DVB-VP) samples were crushed after undergoing significant deformation and flattening under the action of mechanical pinch force. In contrast, the high-crosslinked HCP(St-DVB-VP) sample showed no obvious deformation before crushing, presenting a rigid brittle characteristic. The SEM images of the material surface in Figure 2B demonstrate that the uncrosslinked P(St-DVB-VP) sample has a relatively even surface, and the HCP(St-DVB-VP) sample has a surface structure with distinctly visible openings. The cross-section microstructural images of the materials in Figure 2C show that the porous structure of HCP(St-DVB-VP) is more prominent than that of P(St-DVB-VP).

**FIGURE 3:**
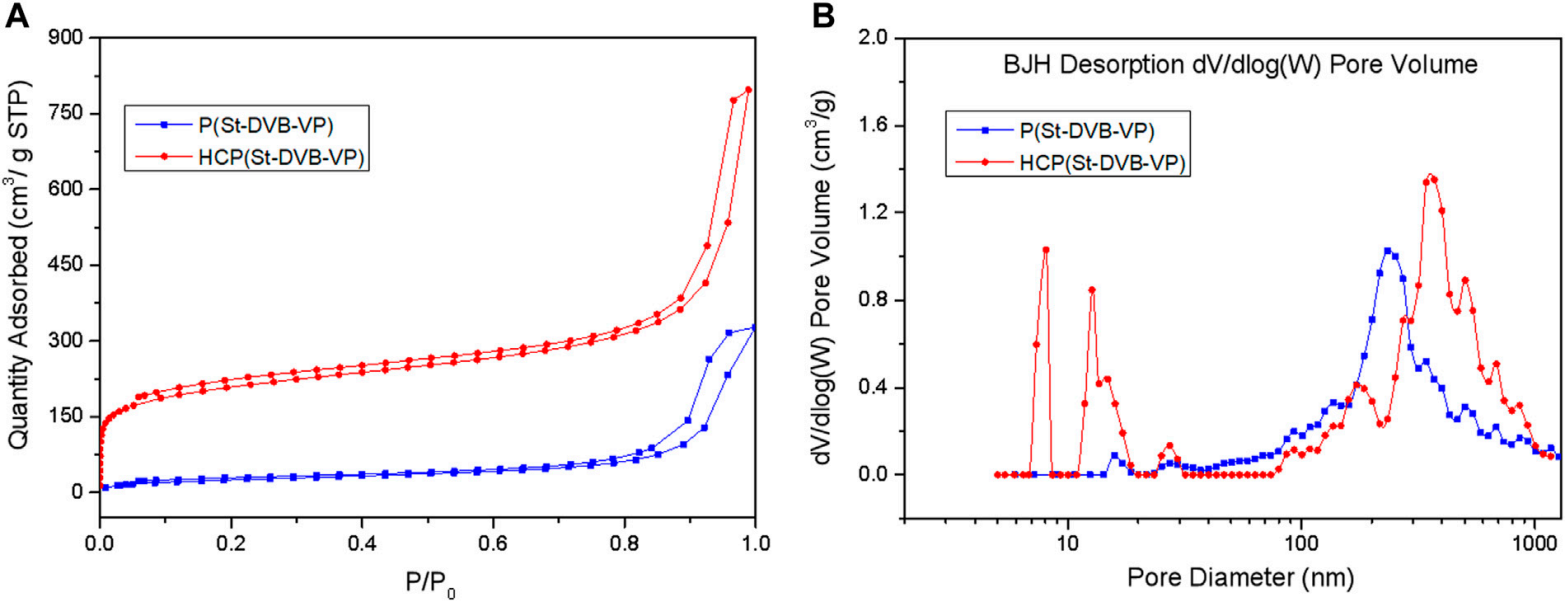
**(A)** N_2_ adsorption-desorption isotherms and **(B)** pore size distribution (PSD) of P(St-DVB-VP) and HCP(St-DVB-VP).

Changes in the porous structure of the adsorptive resin during the post-crosslinking reaction were characterized using Brunauer-Emmett-Teller (BET) nitrogen (N_2_) adsorption-desorption isotherms (Figure 3 and Table 1). As shown by the N_2_ adsorption-desorption isotherms in Figure 3A, the amount of adsorption by HCP(St-DVB-VP) exhibited a sharp rise in the low-pressure zone, indicating the existence of a large number of micropores in the resin. Afterwards, the adsorption amount slowly increases and forms a clear hysteresis loop in the high-pressure zone, indicating the presence of mesopores and macropores in the three-dimensional network structure (Mai et al., 2018). The pore size distribution curves in Figure 3B show that the P(St-DVB-VP) sample does not have micropores and mesopores. In contrast, HCP(St-DVB-VP) contains pores with sizes ranging from 0 to 10 nm, as well as a large number of crosslinking-caused irregular mesopores/macropores, indicating that HCP(St-DVB-VP) has a three-dimensional multilevel (hierarchical) nanonetwork structure.

**TABLE 1:** Textural properties of P(St-DVB-VP) and HCP(St-DVB-VP) samples.

Sample	S_BET_ (m^2^ g^−1^)	S_micro_ (m^2^ g^−1^)	V_p_ (cm^3^ g^−1^)	V_micro_ (cm^3^ g^−1^)	Average pore size (nm)
P(St-DVB-VP)	94	11	0.33	0.0035	63.96
HCP(St-DVB-VP)	761	513	0.74	0.23	7.88

Furthermore, the data in Table 1 show that the HCP(St-DVB-VP) resin derived after the post-crosslinking reaction has a structure with abundant micropores and high specific surface area. The total specific surface area and microporous specific surface area of HCP(St-DVB-VP) are up to 761 m^2^ g^−1^ and 513 m^2^ g^−1^, respectively, which are much higher than those of P(St-DVB-VP). This difference is mainly because the crosslinking agent TMOF increases the crosslinking bridge in the resin during post-crosslinking, which makes the structure of the resin more abundant and stable. There is thus a consequent decrease in the average pore diameter and an increase in the pore volume of HCP(St-DVB-VP), which has an average pore diameter of 7.88 nm and pore volume of 0.74 cm^3^ g^−1^. The above results demonstrate that HCP(St-DVB-VP) with abundant porous structures was successfully obtained using the post-crosslinking method with the crosslinking agent TMOF.

### 3.3 Chemical Structural Characterization of the Adsorptive Resin

**FIGURE 4:**
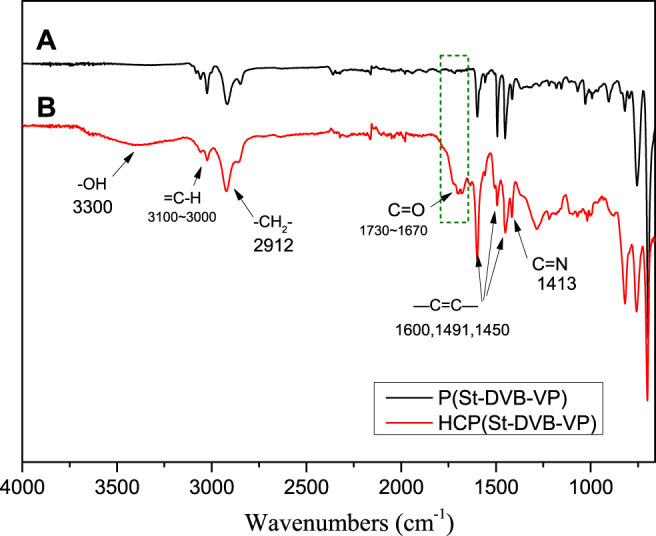
FTIR spectra of **(A)** P(St-DVB-VP) and **(B)** HCP(St-DVB-VP).

Figure 4 shows the FTIR spectra of the before-crosslinking P(St-DVB-VP) and after-crosslinking HCP(St-DVB-VP). As seen from the figure, all samples have stretching/vibration-induced energy absorption peaks at 1,450 cm^−1^, 1,491 cm^−1^, and 1,600 cm^−1^ that belong to the unsaturated C=C of the aromatic ring of the polymeric monomers (Tank and Gupta, 2009); a peak at 3,100–3,000 cm^−1^ that belongs to the unsaturated = C-H; and a peak at 2,912 cm^−1^ that belongs to -CH_2_- on the main chain. The peak at 1,413 cm^−1^ could be assigned to the C=N stretching vibration from pyridine ring of VP monomer (Wu et al., 2003). Compared with P(St-DVB-VP), the HCP(St-DVB-VP) sample has a stretching/vibration-induced energy absorption peak at 3,300 cm^−1^ that belongs to hydroxyl (-OH) and a characteristic peak at 1730–1,670 cm^−1^ that belongs to the carbonyl functional group (C=O) (Tsyurupa et al., 2012). The appearance of these absorption peaks indicates that the P(St-DVB-VP) sample has undergone chemical crosslinking under the action of the small-molecule crosslinking agent TMOF, and new functional groups have been introduced into the product HCP(St-DVB-VP).

**FIGURE 5:**
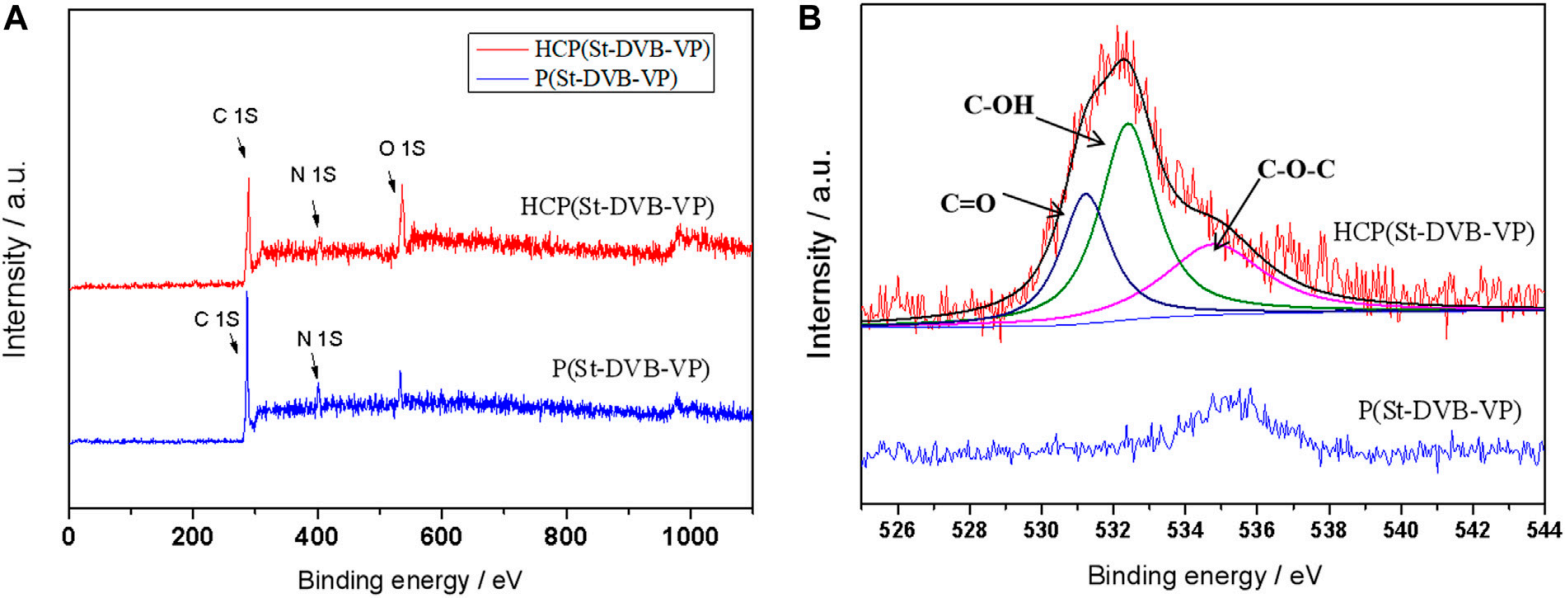
**(A)** Survey XPS spectra and **(B)** O_1s_ XPS spectra of P(St-DVB-VP) and HCP(St-DVB-VP).

Figure 5 presents the X-ray photoelectron spectroscopy (XPS) spectra of the samples before and after crosslinking. As seen from the full spectra in Figure 5A, both samples have obvious *C*_*1s*_ and *N*_*1s*_ peaks. The spectra of the before-crosslinking P(St-DVB-VP) and after-crosslinking HCP(St-DVB-VP) both display a peak at 399.8 eV that belongs to the N atom of the pyridine group (Chen et al., 2017b), indicating that the pyridine group remains in the chemical structure of the resin before and after crosslinking. The spectrum of HCP(St-DVB-VP) after post-crosslinking clearly shows an O_1s_ peak, which was then fitted for band separation. As Figure 5B shows, the characteristic peaks of O atoms in C=O, -OH, and C-O-C appear at 531.1, 532.3, and 535.0 eV, respectively (Moulder et al., 1992), indicating that a new oxygen-containing functional group structure is introduced into HCP(St-DVB-VP) through the post-crosslinking reaction.

### 3.4 Toxin-Adsorbing Properties of Adsorptive Resins in a Plasma Environment

**FIGURE 6:**
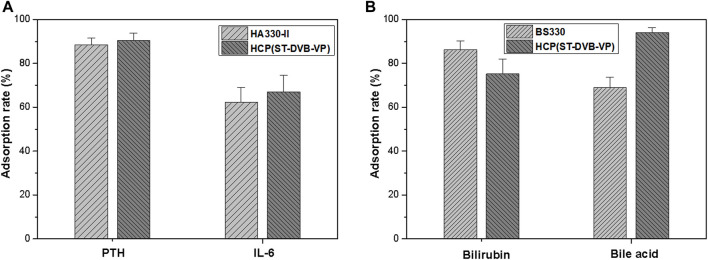
Adsorption rates of **(A)** medium-sized molecular uremic toxins and **(B)** protein-bound toxins in the human plasma after 2 h of treatment. The initial concentrations of PTH, IL-6, bilirubin, and bile acid were 180 pmol L^−1^, 300 pg ml^−1^, 300 μmol/L and 150 μmol/L, respectively. The data are expressed as the mean ± SD of three independent measurements.

Using human plasma as an adsorption environment to simulate a clinical treatment scenario, this study evaluated the ability of the materials to adsorb medium and large molecular toxins (e.g., IL-6) and protein-bound toxins (e.g., bilirubin). As Figure 6A shows, after 2 h of contact adsorption, the adsorption rates of HCP(St-DVB-VP) towards IL-6 and PTH are 67.1 ± 7.6% and 90.5 ± 3.3%, respectively, which were slightly higher than that of HA330-Ⅱ (IL-6: 62.2 ± 7.0%, PTH: 88.5 ± 3.0%). In Figure 6B, after 2 h of contact adsorption in the plasma environment, HCP(St-DVB-VP) has an adsorption rate of 75.2 ± 7.0% and 94.1 ± 2.2% towards bilirubin and bile acid, respectively, showing adsorption capacities equivalent to that of the existing commercial BS330 resin in perfusion devices. This result confirms that HCP(St-DVB-VP) has a satisfactory capacity to adsorb protein-bound toxins. According to results of calculation, the adsorbed mass of PTH, IL-6, bilirubin and bile acid to HCP(St-DVB-VP) reached about 6.52 pmol/g, 8.05 ng/g, 9.02 μmol/g and 5.65 μmol/g, respectively. In conclusion, the HCP(St-DVB-VP) resin prepared in this study showed an excellent performance in adsorbing both medium and large molecular toxins and protein-bound toxins.

The ability of HCP(St-DVB-VP) to effectively adsorb different toxins can be attributed to its abundant porous structure and surface chemical properties. Some studies have found that one of the conditions conducive to adsorption is that the sizes of the transport channels and adsorption channels of the adsorptive resin are more than 10 times and 2 times the size of the material to be adsorbed, respectively. The molecular sizes of IL-6 and PTH are approximately 4.632 and 3.018 nm, respectively. The porous structural data show that HCP(St-DVB-VP) has a three-dimensional multilevel (hierarchical) nanonetwork structure and an average pore size of 7.88 nm, which is larger than the molecular sizes of the target substances. The macropores can act as toxin-transporting channels, facilitating adsorption and transport of toxins in the resin. A large number of mesoporous and microporous structures in the resin act as adsorption channels for the resin to adsorb and capture toxins, enabling the resin to achieve a satisfactory adsorption performance (Harm et al., 2014).

Bilirubin, an endogenous protein-bound uremic toxin (PBUT), has a molecular weight of only 584. However, it mainly exists in the form of a bilirubin-albumin complex in plasma, and only a small amount of free bilirubin exists in plasma (Greige-Gerges et al., 2007). Therefore, generally, bilirubin cannot be effectively removed through hemoperfusion with common macroporous adsorptive resins. The HCP(St-DVB-VP) resin obtained in this study, in addition to its abundant porous texture, has pyridine functional groups on the surface. The pyridine groups, which have a certain amount of positive charge, can interact with the negatively charged bilirubin. In addition, the pyridine groups can promote bilirubin liberation from the “bilirubin-albumin complex”, and then, the resultant free bilirubin is further adsorbed by the resin, eventually reaching equilibrium and achieving relatively strong bilirubin adsorption.

### 3.5 Adsorption Isotherms and Kinetics Towards Toxins in a PBS Environment

To better understand the adsorption capacity and characteristics of the adsorptive resin towards protein-bound toxins and medium/large-sized molecular toxins, bilirubin and lysozyme in PBS were used as model molecules to investigate the adsorption behaviour of HCP(St-DVB-VP) towards different toxins. Due to the inability to obtain sufficient medium/large-sized molecular toxins in the human body, lysozyme (MW = 14 kDa) is often used as a model to evaluate the removal performance of hemoperfusion materials for the removal of medium/large-sized molecular toxins (Chen et al., 2017c).

**FIGURE 7:**
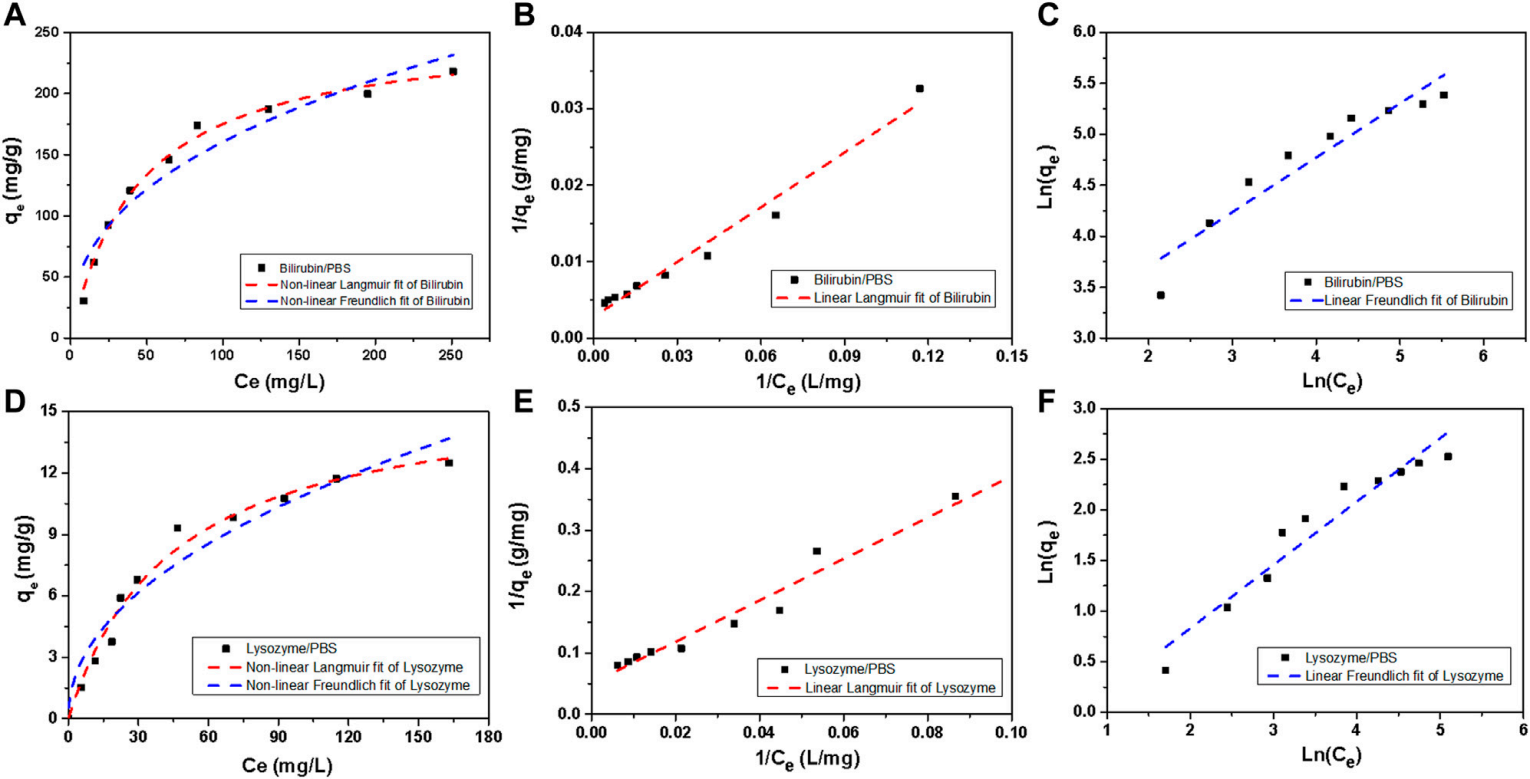
Bilirubin and lysozyme adsorption isotherms of HCP(St-DVB-VP). **(A,D)** Non-linear Langmuir and non-linear Freundlich, **(B,E)** linear Langmuir, and **(C,F)** linear Freundlich model fitting plots.

Figure 7 shows the adsorption isotherms of HCP(St-DVB-VP) towards bilirubin and lysozyme in PBS. Linear and nonlinear Langmuir (Eqs 4, 5) and Freundlich (Eqs 6, 7) isothermal adsorption models were used for fitting (Foo and Hameed, 2010; Li et al., 2020). The fitting results are shown in Figure 7 and Table 2.

**TABLE 2:** Adsorption isotherm parameters of HCP(St-DVB-VP) towards bilirubin and lysozyme.

Toxin	Fitting method	Langmuir			Freundlich		
		*q*_*m*_ (mg g^−1^)	*K*_*L*_ (L mg^−1^)	*R* ^*2*^	*K*_*F*_ (mg g^−1^) (L mg^−1^)^1/n^	1/*n*	*R* ^*2*^
Bilirubin	Linear	357.14	0.01173	0.9740	14.0236	0.53257	0.8935
	Non-linear	254.11	0.02219	0.9906	25.6741	0.39813	0.9224
Lysozyme	Linear	19.66	0.01510	0.9884	0.65687	0.62563	0.9227
	Non-linear	16.17	0.02261	0.9765	1.23903	0.47147	0.9205

The nonlinear Langmuir isothermal adsorption formula is as follows:

qe=qmKLCe(1+KLCe). (4)

The linear Langmuir isothermal adsorption formula is as follows:

1qe=1KLqmCe+1qm. (5)

The nonlinear Freundlich isothermal adsorption formula is as follows:

$$q_{e}\;=\;K_{F}C_{e}^{1/n}.$$ (6)

The linear Freundlich isothermal adsorption formula is as follows:

ln(qe)=1nln(Ce)+ln(KF). (7)

In the formulas, *q*_*e*_ is the equilibrium adsorption amount (mg g^−1^); *q*_*m*_ is the maximum adsorption amount (mg g^−1^); *Ce* is the concentration of toxin at adsorption equilibrium (mg L^−1^); *K*_*L*_ is the Langmuir adsorption equilibrium constant (L mg^−1^); *K*_*F*_ is the Freundlich constant ((mg g^−1^) (L mg^−1^)^1/*n*^), which reflects the adsorption capacity of the adsorbent; *n* is the adsorption intensity of the adsorbent, which can be used to judge the difficulty of the reaction.

As Table 2 shows, in all cases, the degree of fitting *R* ^*2*^ (>0.97) obtained using the Langmuir isothermal adsorption model (hereinafter abbreviated as the Langmuir model) is higher than that of the Freundlich isothermal adsorption model (0.89–0.92), indicating that the Langmuir model is more suitable to describe HCP(St-DVB-VP) adsorption of bilirubin and lysozyme. Generally, the Langmuir model assumes that the adsorption is a monolayer process so that the adsorption reaches its maximum when saturation is reached on the adsorbent surface. In other words, the adsorption of bilirubin and lysozyme by HCP(St-DVB-VP) is a monolayer surface adsorption process, and their adsorption reaches saturation when adsorption on the resin surface reaches its maximum. According to the fitting results of the nonlinear Langmuir model, the maximum adsorption capacity of HCP(St-DVB-VP) towards bilirubin and lysozyme can reach 357.14 mg g^−1^ and 19.66 mg g^−1^, respectively. In addition, Figure 7 and Table 2 show that the Freundlich adsorption model also has a relatively satisfactory performance, as evidenced by the results that both the *K*_*F*_ and *n* values are larger than 1. This finding indicates that HCP(St-DVB-VP) has a strong affinitive adsorption capacity towards bilirubin and lysozyme (Zeng et al., 2009).

**FIGURE 8:**
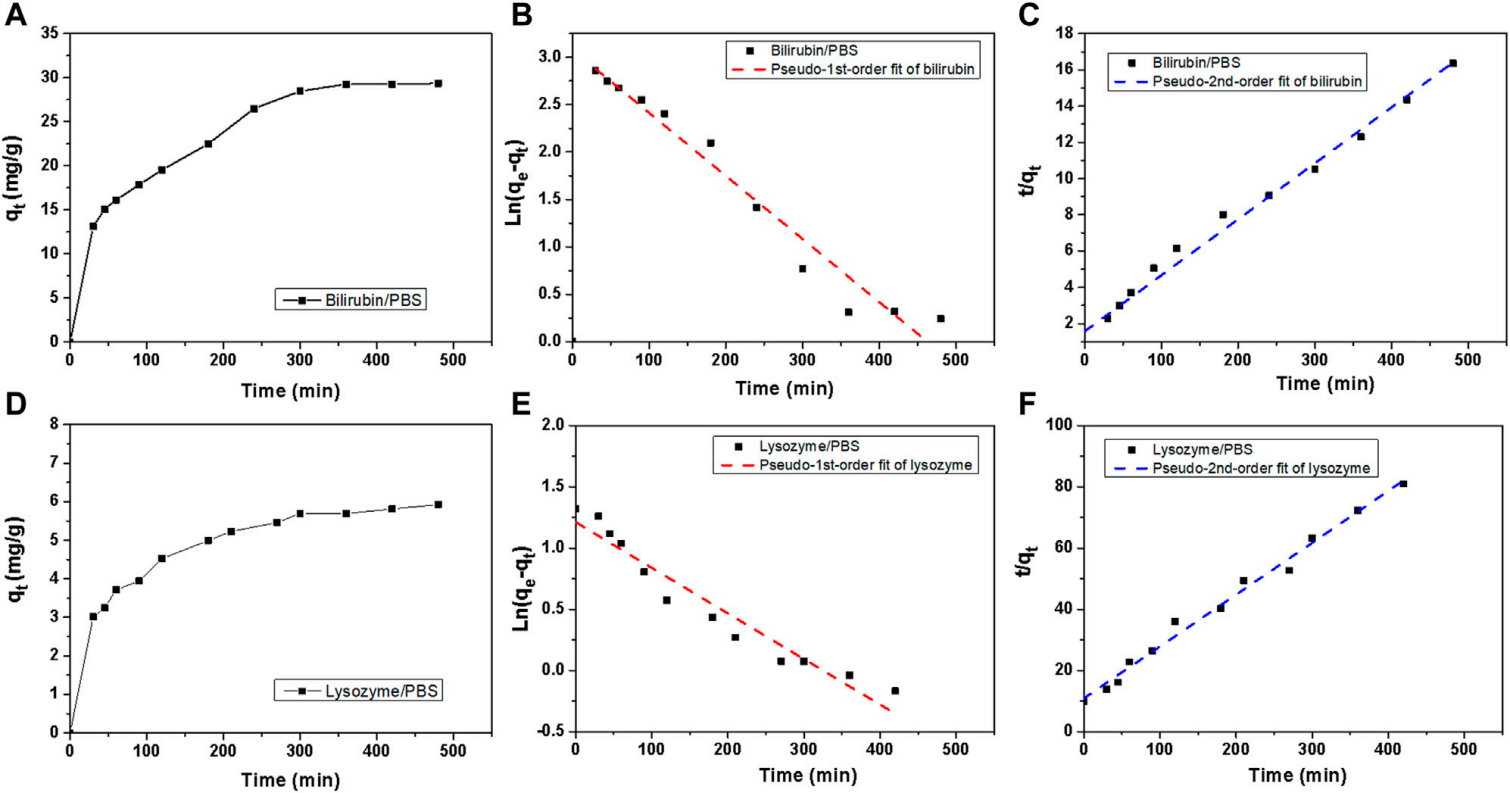
Bilirubin and lysozyme adsorption kinetics of HCP(St-DVB-VP). **(A,D)** Toxin uptake versus times in PBS. **(B,E)** Adsorption kinetic pseudo-1st-order and **(C,F)** pseudo-2nd-order fitting plots for bilirubin adsorption.

To further investigate the adsorption kinetics of HCP(St-DVB-VP) towards bilirubin and lysozyme, an adsorption experiment was performed at 37°C, which simulates human body conditions, using bilirubin- and lysozyme-containing PBS solutions. The toxin residual concentration in the solution at different time points was measured to calculate the change in the adsorption amount qt with time. As shown in Figure 8A, the adsorption of bilirubin and lysozyme by HCP(St-DVB-VP) was relatively fast, reaching a high peak at approximately 120 min; afterwards, the adsorption shows a slow upwards trend over time.

The adsorption rate of the solute by the resin can be described by the kinetics of the adsorption process. According to the data for bilirubin and lysozyme adsorption by HCP(St-DVB-VP), a quasi-first-order kinetic equation (Eq. 8) and quasi-second-order kinetic equation (Eq. 9) were used to fit the adsorption process (Ho and McKay, 1999; Tao et al., 2014).

$$\ln\left(q_{e}-q_{t}\right)=\ln q_{e,cal,1}-k_{1}t,$$ (8)

tqt = 1k2qe,cal,22 + tqe,cal,2. (9)

In the formulas, *q*_*e*_ and *q*_*t*_ are the amount of toxin adsorbed by HCP(St-DVB-VP) at the time of adsorption equilibrium and t (mg g^−1^), respectively; and *q*_*e*,__*cal*,1_ and *q*_*e*,__*cal*,2_ are the equilibrium adsorption amounts (mg g^−1^) calculated using the fitting formulas. *K*_*1*_ is the adsorption rate constant (min^−1^) of the quasi-first-order equation, which was determined based on the slope fitted from the plot of ln (*q*_*e*_−*q*_*t*_) against *t*. *K*_*2*_ is the adsorption rate constant (g mg^−1^ min^−1^) of the quasi-second-order equation; and *q*_*e*,__*cal*, 2_ and *K*_*2*_ were determined from the slope and intercept, respectively, of the plot of *t*/*q*_*t*_ against *t*.

Figures 8B,C show the fitting curves of the quasi-first-order and quasi-second-order equations for adsorption kinetics, respectively, and Table 3 presents the fitting parameters. In all cases, the *R* ^*2*^ value of fit obtained from the second-order kinetic equation is larger than that from the first-order equation and shows a good linear relationship (Figure 8C). The equilibrium adsorption capacity (*q*_*e*_) obtained by the fitting of the second-order equation for adsorption kinetics of bilirubin and lysozyme is 32.38 mg g^−1^ and 5.9 mg g^−1^, respectively, which are close to the results of 30.63 mg g^−1^ and 6.77 mg g^−1^ obtained from experiments (Table 3). This result indicates that the quasi-second-order fitting equation for kinetics is more appropriate for describing the process of bilirubin and lysozyme adsorption by HCP(St-DVB-VP) resin. Thus, the adsorption of bilirubin and lysozyme by HCP(St-DVB-VP) is affected by both concentration and active sites.

**TABLE 3:** Adsorption kinetic parameters of HCP(St-DVB-VP) towards bilirubin and lysozyme.

PBUT	*q*_*e*,exp_ [mg g^−1^]	Pseudo-1st-order			Pseudo-2nd-order		
		*q*_*e*,cal,1_ [mg g-1]	*k*_*1*_ [min^−1^]	*R* ^*2*^	*q*_*e*,*cal*,2_ [mg g-1]	*k*_*2*_ [g^−1^ mg^−1^ min^−1^]	*R* ^*2*^
Bilirubin	30.63	21.73	0.00665	0.9580	32.38	0.0006	0.9812
Lysozyme	6.77	3.36	0.00373	0.9358	5.90	0.0026	0.9881

### 3.6 Hemocompatibility of Adsorptive Resin

**FIGURE 9:**
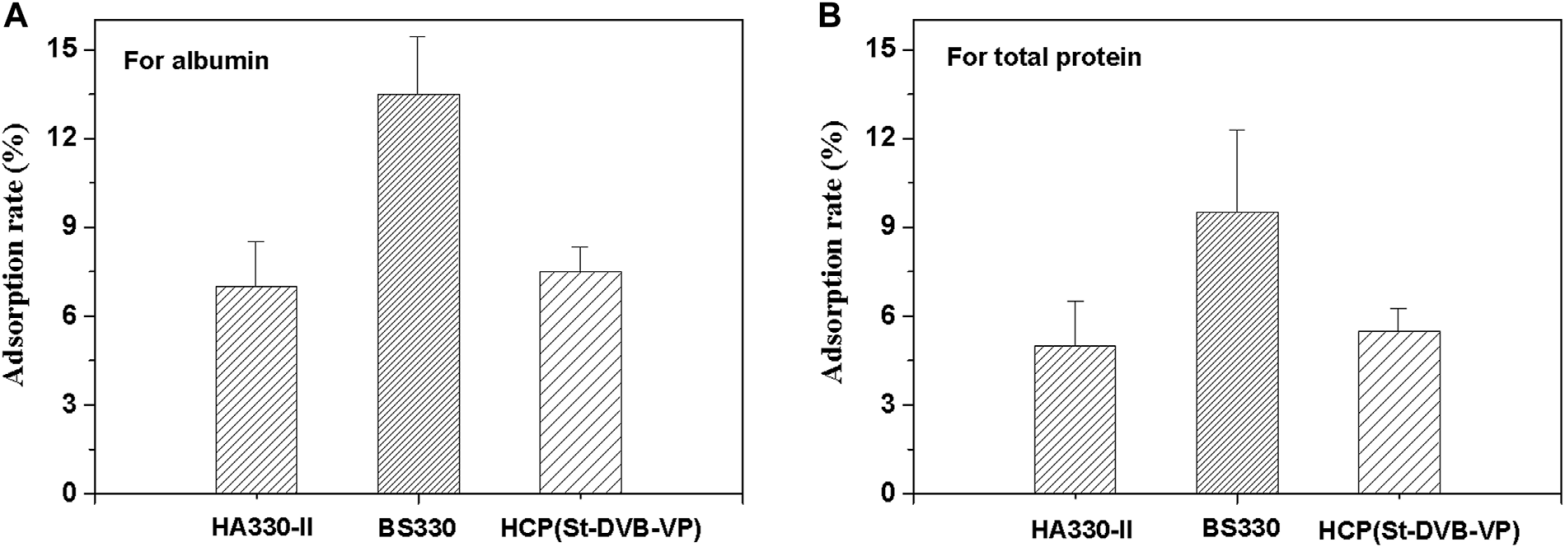
Calculated adsorption rates of the **(A)** albumin and **(B)** total protein from human plasma on various adsorbents. All values are expressed as the mean ± SD of three independent measurements.

In medical applications, a critical requirement for hemoadsorbent materials is hemocompatibility. Plasma protein adsorption is the first reaction when blood interfaces with an artificial surface. An ideal blood perfusion adsorbent should effectively resist non-specific protein adsorption. In this work, the adsorption of human plasma albumin, human plasma total protein to absorbents were investigated, and the results were presented in Figure 9. The calculated albumin adsorption rates of HCP(St-DVB-VP) and HA330-II were 7.5 ± 0.8% and 7.0 ± 1.5%, respectively, and there was no significant difference between the two groups. BS330 had a albumin adsorption rate of 13.5 ± 2.0%. The calculated total protein adsorption rates for HA330-Ⅱ, BS330 and HCP(St-DVB-VP) were 5.0 ± 1.5%, 9.5 ± 2.8% and 5.5 ± 0.9%, respectively. According to these results, the HCP(St-DVB-VP) exhibited similar anti-protein nonspecific adsorption performance compared to HA330-II adsorbent, and BS330 showed the worst protein adsorption resistance.

**FIGURE 10:**
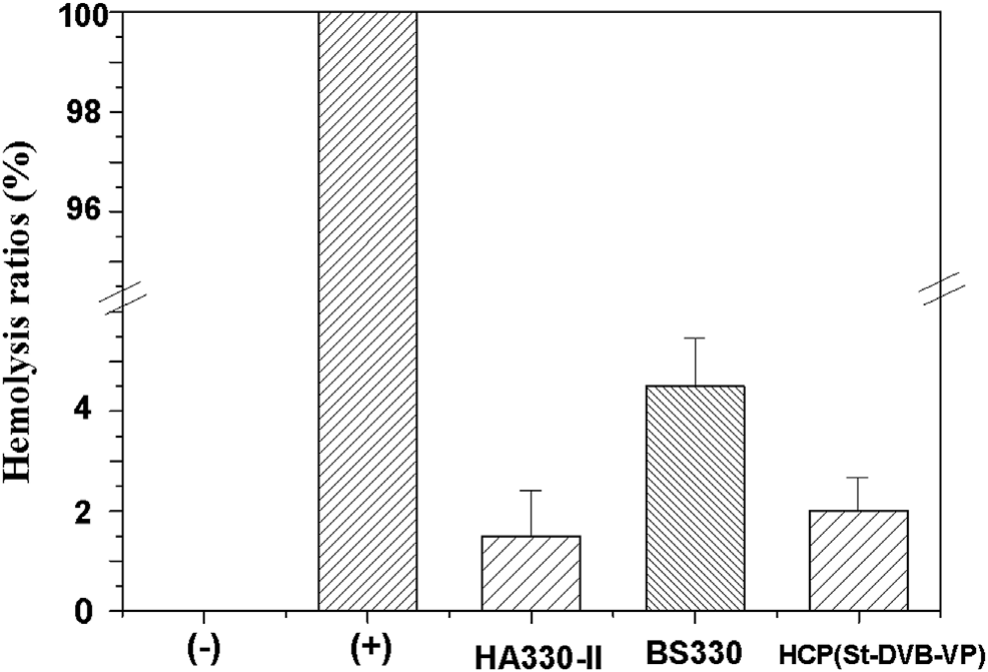
Hemolysis ratio of the controls and adsorbents. hemolysis test: (+) deionized water, (−) PBS solution. The data are expressed as the mean ± SD of three independent measurements.

During hemoperfusion therapy, the unavoidable direct contact of the adsorptive resin with the blood may cause safety issues if the material-induced hemolysis exceeds a specified limit. Therefore, hemolysis assays assessing erythrocyte lysis and free hemoglobin are usually used to sensitively evaluate the impact on erythrocytes and the hemocompatibility of adsorptive materials (Gu et al., 2013). Figure 10 displays the results from hemolysis assays of the materials in this study. The hemolysis rate of BS330 is 4.5 ± 1.0%, which is relatively high. The hemolysis rate of the HCP(St-DVB-VP) adsorbent is 2 ± 0.6%, which is the same level as the existing commercial whole blood perfusion adsorbent resin HA330-II (1.5 ± 0.9%). Both HCP(St-DVB-VP) and HA330-II have a hemolysis rate lower than the specified limit and meet the ISO10993-4:2002 requirement that the hemolysis rate of medical products should not be more than 5%. The results indicate that the HCP(St-DVB-VP) adsorbent causes only minor damage to erythrocytes and demonstrates high hemocompatibility.

**FIGURE 11:**
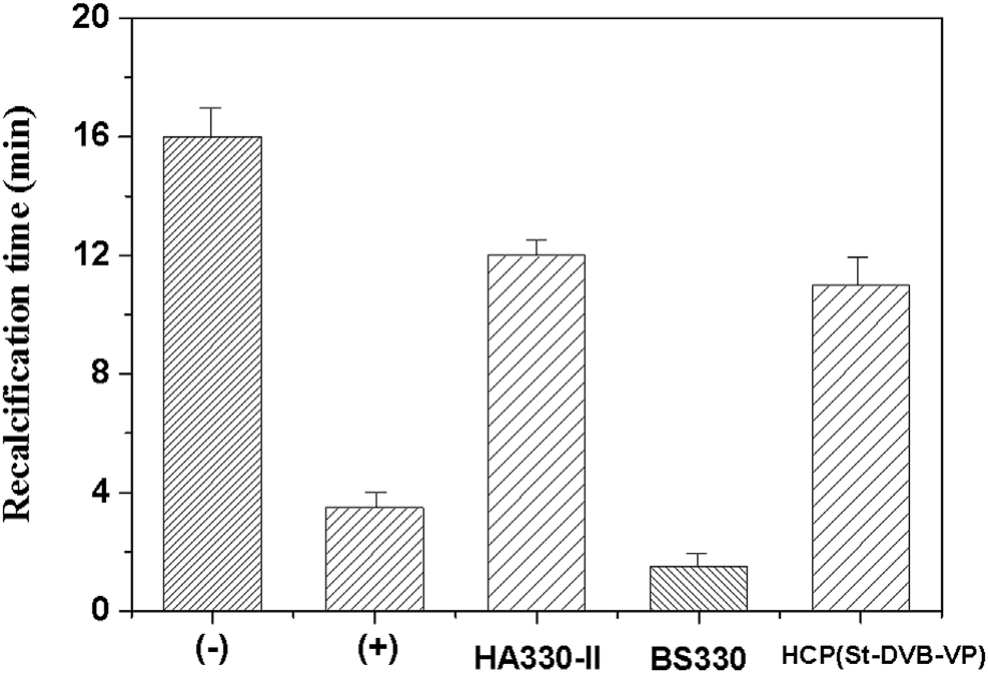
Recalcification time of the controls and adsorbents. (+) Hydrophilic glass beads, (−) blank tube. The data are expressed as the mean ± SD of three independent measurements.

Further, a “recalcification time” experiment was conducted with PRP to evaluate the effect of adsorptive resin on coagulation activation. This method can simultaneously detect the activation of prothrombin and platelets caused by the material to be tested and has a high sensitivity (Liu et al., 2021). In the recalcification time experiment, the coagulation factor FXII and platelets in plasma are activated to varying degrees after the plasma is incubated with the material. Addition of calcium chloride solution initiates the endogenous coagulation pathway and triggers conversion of a large amount of plasma fibrinogen to fibrin, which coagulates the plasma into a gel. The higher the degree of activation when the plasma is in contact with the material, the shorter the plasma clotting time. The results of this experiment (Figure 11) revealed that the plasma incubated with the resin sample became coagulated sooner. The gel appeared 3–4 min after the start of timing, and the plasma quickly coagulated. In contrast, the negative control, i.e., the plasma without incubation with the resin sample, had a coagulation time of 16–17 min. The BS330 resin had the shortest recalcification time, and plasma coagulation occurred within approximately 1.5 ± 0.5 min, indicating that BS330 can significantly activate PRP. This finding may be attributed to a high level of platelet activation by BS330, which also means that BS330 is not suitable for whole blood perfusion therapy. The recalcification time of HCP(St-DVB-VP) and HA330-II was 11.0 ± 1.0 min and 12.0 ± 0.5 min, respectively. These results suggested that the coagulation effects of HCP(St-DVB-VP) and HA330-Ⅱ were close to that of negative control, and both of them exhibited low clotting risk.

## 4 Conclusion

In this study, a pyridinyl-modified HCP porous resin adsorbent, HCP(St-DVB-VP), was successfully prepared through a Friedel-Crafts post-crosslinking reaction using trimethyl orthoformate as crosslinking agent for the first time. The resin had a three-dimensional multilevel (hierarchical) nanonetwork structure, and its total specific surface area and micropore specific surface area were up to 761 m^2^ g^−1^ and 513 m^2^ g^−1^, respectively. Adsorption experiments showed that HCP(St-DVB-VP) exhibited an excellent adsorptive property towards both protein-bound toxins and medium and large molecular toxins. In human plasma, the adsorption rates of HCP(St-DVB-VP) towards IL-6 and PTH were 67.1 and 90.5%, respectively, slightly higher than those of HA330-II resin. The adsorption rates towards bilirubin and bile acid reached 75.2 and 94.1%, respectively, which were similar to those of the existing commercial perfusion resin BS330. The thermodynamic isotherms, kinetic curves, and simulation analysis revealed that the process of HCP(St-DVB-VP) adsorption of bilirubin and lysozyme can be appropriately described using a quasi-second-order kinetic equation, in particular, the Langmuir isothermal adsorption model. Thus, the adsorption of toxin by the resin was a monolayer surface adsorption process controlled by chemical adsorption. In addition, based on the blood compatibility assessments in this study, HCP(St-DVB-VP) exhibited low hemolysis rate and low clotting risk, demonstrating acceptable hemocompatibility, which was similar to HA330-II.

The preparation method for the multi-functional HCP resin developed in this study is green and environmentally friendly. The as-prepared resin can adsorb and remove both protein-bound toxins and medium- and large-sized molecular toxins, exhibiting a broad adsorption effect. Thus, HCP(St-DVB-VP) is a promising adsorbent can be used in clinical whole blood perfusion in artificial liver blood purification.

## Data Availability

The original contributions presented in the study are included in the article/Supplementary Material, further inquiries can be directed to the corresponding author.
